# Positioning the National Health Insurance for financial sustainability and Universal Health Coverage in Ghana: A qualitative study among key stakeholders

**DOI:** 10.1371/journal.pone.0253109

**Published:** 2021-06-15

**Authors:** Moses Aikins, Philip Teg-Nefaah Tabong, Paola Salari, Fabrizio Tediosi, Francis M. Asenso-Boadi, Patricia Akweongo

**Affiliations:** 1 Department of Health Policy Planning and Management, School of Public Health, University of Ghana, Legon, Accra; 2 Department of Social and Behavioural Sciences, School of Public Health, University of Ghana, Legon, Accra; 3 Swiss Tropical and Public Health Institute, Basel, Switzerland; 4 University of Basel, Basel, Switzerland; 5 National Health Insurance Authority, Accra, Ghana; University of California San Francisco, UNITED STATES

## Abstract

**Introduction:**

The National Health Insurance Scheme (NHIS) was introduced in 2003 to reduce “out-of-pocket” payments for health care in Ghana. Over a decade of its implementation, issues about the financial sustainability of this pro-poor policy remains a crippling fact despite its critical role to go towards Universal Health Coverage. We therefore conducted this study to elicit stakeholders’ views on ways to improve the financial sustainability of the operations of NHIS.

**Methods:**

Twenty (20) stakeholders were identified from Ministry of Health, Ghana Health Services, health workers groups, private medical practitioners, civil society organizations and developmental partners. They were interviewed using an interview guide developed from a NHIS policy review and analysis. All interviews were recorded and transcribed verbatim. The data were analysed thematically with the aid of NVivo 12 software.

**Results:**

Stakeholders admitted that the NHIS is currently unable to meet its financial obligations. The stakeholders suggested first the adoption of capitation as a provider payment mechanism to minimize the risk of providers’ fraud and protection from political interference. Secondly, they indicated that rapid releases of specific statutory deductions and taxes for NHIS providers could reduce delays in claims’ reimbursement which is one of the main challenges faced by healthcare providers. Aligning the NHIS with the Community-based Health Planning and Services and including preventive and promotive health is necessary to position the Scheme for Universal Health Coverage.

**Conclusion:**

The Scheme will potentially achieve UHC if protected from political interference to improve the governance and transparency that affects the finances of the scheme and the expansion of services to include preventive and promotive services and cancers.

## Introduction

Social protection plays an important role in preventing as well as reducing poverty in all societies [1,2]. The first United Nations Sustainable Development Goal envisions to eradicate poverty of all forms across member countries. Closely related to this is the global agenda of universal access to health care which is espoused in several international conventions and treaties [3]. Recognizing the nexus between poverty and access to health services has formed the basis for integrated programmes across the world [4–7].

Access to quality health care is a human right issue and has been articulated in many local and international treaties [8,9]. Governments in most low-income countries are therefore grappling with the challenges associated with providing high quality services and strengthen the health system [10,11]. The health system has been defined as all the institutions with the primary purpose of promoting, restoring or maintaining health [12]. The World Health Organization (WHO) identified six building blocks that are relevant in strengthening the health system to make it efficient and effective [13]. Health financing is one of the critical building blocks of the health system. Reducing financial barrier to health care is essential in achieving Universal Health Coverage (UHC). As a result, many countries have developed insurance schemes to reduce the catastrophic effects of out-of-pocket payments [14].

Ghana is one of the few countries in sub-Saharan Africa that took an early lead and introduced a nationwide health insurance program [15,16]. Ghana introduced the National Health Insurance Scheme (NHIS) to replace the cash and carry system which was restricting accessibility to health services. In this Scheme, subscribers with valid membership are eligible for free treatment when needed. The law that established the Scheme clearly indicates that the NHIS should be financed through a central National Health Insurance Fund (NHIF) which is sourced from the National Health Insurance Levy (NHIL) of 2.5% tax on selected goods and services; 2.5% of Social Security and National Insurance Trust (SSNIT) contributions, largely by formal sector workers, government budgetary allocations, grants, donations, and proceeds of investments made by the national health insurance council [17]. Membership of the insurance scheme by the informal sector is through premium contributions and periodic renewal. Persons below 18 or above 70 years of age, SSNIT pensioners, pregnant women, or persons deemed indigent are exempt from premium payments [18]. The levy (NHIL) is collected by the Domestic Tax Revenue Division of the Ghana Revenue Authority through VAT-registered persons in the same way that VAT is collected. The Ghana Revenue Authority (GRA) is expected to pay the collected levy directly into the National Health Insurance Fund within thirty days of collection [19].

The Scheme cover about 95% of common diseases in the country [20].The NHIS benefit package includes outpatient and inpatient services, essential drugs, inpatient accommodation, and maternity care including cesarean delivery, dental care, eye care, and emergency care with provision for inclusion of other services in future [21]. Under the National Health Insurance Scheme (NHIS), healthcare services covered under the minimum benefit package are delivered by credentialed service providers. From the inception of NHIS, the National Health Insurance Authority (NHIA) employed fee-for-service (FFS) method for the payment of its credentialed providers. FFS is a payment model where services are unbundled and paid for separately [22]. However, the scheme later observed challenges in processing claims expenditure because of increasing utilization. To ameliorate the situation, the NHIA introduced the diagnosis-related-grouping (DRG) payment method [23]. Under DRGs, providers are reimbursed at a fixed rate per service based on diagnosis and treatment. In 2012, the NHIA introduced the capitation system of payment for primary out-patients’ services on a pilot basis in the Ashanti Region of Ghana. With capitation scheme, providers are paid a fixed amount of money on the basis of number of patients for delivering a range of services. However, this could not be scaled up because service providers opposed the system [24].

Since the implementation of the Scheme, it has been reported that it has contributed to the increase in access to health care. Witter and Garshong noted that the number of outpatient visits per capita in Ghana increased sharply after 2005, the same year NHIS operations began [20]. It has also been reported that pregnant women enrolled in the NHIS are more likely to receive prenatal care, give birth in a hospital, and have skilled attendants present at birth [25]. An earlier study conducted in Ghana also found that there has been an increase in health care services utilisation which is attributable to the introduction of the NHIS [26,27]. It has also been acclaimed as Ghana’s ultimate strategy for achieving UHC [28,29]. However, over a decade of its implementation, the NHIS is still faced with major obstacles that are likely to undermine its success and sustainability. One such obstacle that threaten the operations of the Scheme is financial sustainability. Earlier researchers have raised doubts about the Scheme’s ability to continue to meet its financial obligations [21,30]. This study was therefore conducted to document stakeholders’ perspectives on how to position the Scheme for financial sustainability to ensure Universal Health Coverage.

## Material and methods

### Ethics statement

The protocol for the study was reviewed and approved by the Ghana Health Service Ethics Review Committee (GHS-ERC 01/05/16). Written consent was obtained from all participants in this study. We also used pseudonyms to identify our participants.

### Study design and setting

The study adopted a narrative approach to qualitative enquiry. Narratives permit life-like accounts that focus on experience, hence their alignment with qualitative orientation. To create narratives, the interview data is processed to describe factual information provided by and about the participants from the field texts [31]. Narratives enable participants’ stories and descriptions of experience to be honoured and given status [32]. In adopting this approach, we allowed stakeholders to share their interest in NHIS before responding to our questions. This strategy enabled the researchers to identify personal biases of participants to improve the credibility of the study findings. In conducting the study, we adhered to the consolidated criteria for reporting qualitative research (COREQ) [33], and acceptable practice in fieldwork, analysis and interpretation [34].

The study was conducted in Ghana. In terms of health service delivery, the country operates a five-tiered service system, including: National, Regional, District, Sub-district and the Community [35]. The regional, district and community levels, health facilities are established to provide primary and secondary health care services. In 1999, Ghana adopted the Community-based Health Planning and Services (CHPS) strategy as a primary health care system [36]. In the CHPS strategy, Community Health Officers (CHOs) are assigned to a demarcated CHPS zones as a measure to ensure the close-to-client model of community health services in those demarcated zones [37]. The CHOs provide a door-to-door services with support from community volunteers [38].

Ghana also developed a social insurance system known as the National Health Insurance Scheme. The initial law governing the operations of the Scheme (National Health Insurance Act 650 of 2003) was revised in 2012 to Act 852 [21]. The National Health Insurance Authority is responsible for ensuring access to healthcare services to the persons covered by the Scheme. Several laws and policies have been developed by the government of Ghana to guide the implementation of the health insurance scheme and social protection for the poor and vulnerable in society. These laws and policies formed the basis for the selection of stakeholders.

### Analytical approach and stakeholders’ identification

The stakeholder analysis was guided by some key questions: Legitimacy (does the stakeholder hold an influential position with strong legitimacy?); resources (does the stakeholder have material and immaterial resources to influence policy?); network (is the stakeholder well connected with other influential stakeholders?), influence and power (does the stakeholder exercise influence and power to facilitate or impede reforms in the NHIS?). Based on these issues, stakeholders were classified into two groups: the first group included the key stakeholders, likely to influence the NHIS policy; the second group included actors in the implementation of the NHIS. Only high-level decision-makers were contacted and interviewed at each of the identified institutions. In all, twenty (20) stakeholders were interviewed for this study (See Supporting information).

### Data collection

We prepared an interview guide prior to conducting semi-structured interviews to ensure that the same basic lines of inquiry were pursued. Participants were encouraged to talk about their experiences with the NHIS, governance, transparency and sustainability issues of importance to them (see Supporting information). We provided inputs to guide the conversation. Similarly, prompts were used to elucidate participants’ background, personal highlights, setbacks and critical incidents in implementation of the NHIS. All the interviews were conducted by a seasoned qualitative expert and researcher. The interviews were all conducted in English at the office of selected participants. It took between 30 to 45 minutes to complete an interview session. All the participants identified as key stakeholders agreed to be interviewed. After each interview session, the audio was replayed to the participant for validation. Stakeholder dissemination meeting was also held for participants. Post-dissemination discussions were conducted to enrich the findings of the study.

### Qualitative data analysis

The qualitative interviews were recorded using audio recorder and transcribed verbatim. We reviewed the transcripts and developed a codebook which served as guide for the thematic analysis [39]. The codebook was developed by one of the authors. Conceptual dimensions of the interview guides were used to develop the preliminary codebook. This was then revised to include the emerging themes from the data. The codebook contained names of the code, brief definition, detailed descriptions, when to use that code and examples of statements that fall within that code. The transcripts were imported into NVivo 12. The code names were used to create nodes in the NVivo. Each transcript was opened in the software coded and reviewed. Initially, coding was done into free nodes. However, as the coding progressed, the relationship between nodes became clearer and the free nodes were transformed into tree nodes. The coding was done by two of the researchers using the codebook. After the coding, a code comparison query was used to determine the level of agreement between the researchers in NVivo. An intercoder reliability index (Kappa coefficient) was computed as 0.89, showing a higher level of agreement [40]. Coded sections were regrouped into relevant categories and themes for presenting the results. Direct quotations were used, where appropriate, to support the themes.

## Results

The themes that emerged from the data were grouped into three hierarchical categories. First the main study areas themes—named global themes–included access to health care, financial sustainability of the scheme, governance and transparency issues. Within each of the “global” themes, the other themes that emerged were classified into main and sub-themes. Table 1 shows the themes and sub-themes that were identified.

**Table 1:** Themes that emerged from the data.

Global themes	Main themes	Sub-themes
Access to health care	Role of NHIS in increasing access	• Improving health care access • Universal Health Coverage
	Inclusion of preventive services	• Aligning NHIS to CHPS • Inclusion of routine screening • Inclusion of telemedicine
Sustainability	Cost containment challenges	• Broad exemptions • Provider Fraud • Low active membership • Mark-ups to cater for delays in re-imbursement
	Increasing funding sources	• New funding sources • Quick releases of NHIL and NHIF • Introduction of special premium
	Claims reimbursement	• Vetting of claims • Capitation • Gatekeeper System
	Expanding enrollment and active membership	• Mandatory requirement for accessing essential services
Governance structure	Governance and transparency	• Relationship between NHIS and Ministry of Health • Credentialing of health facilities • Determining tariffs • Releases of statutory deduction for NHIS • Use of NHIS funds

### Role of NHIS in increasing access to health care and extension of the benefit package

The introduction of the NHIS was seen as the pro-poor policy that took away financial barrier in seeking health care. Participants were unanimous in the critical role the Scheme was playing in the country’s move toward UHC.

*The Scheme has increased access to health care for all categories of Ghanaians*. *It can play an important role in Universal Health Coverage to ensure that everybody has access to health care”* (Stakeholder 20).

Although it was unanimous that NHIS was bridging financial barriers to health care, it was suggested that to position the Scheme for UHC, several stakeholders mentioned the need for the inclusion of prevention services and cancers in the benefit package. The services should include medical examination for members of the scheme. In their opinion medical examination will facilitate early detection of non-communicable diseases such as diabetes, hypertension and cancers. Further, medical examination or screening can improve the health seeking behaviour of scheme members.

*“The NHIS card holders must be allowed to do medical examination two times in a year*. *This will help detect conditions early for management than the reactive (curative) approach”* (Stakeholder 5).

Participants also opined the need for expansion of the benefits package to include preventive and health promotive activities rendered by the Community Health Officers (CHOs) in the Community-based Health Planning and Services (CHPS). These primary health care facilities should therefore be reimbursed by the NHIS for their services. The door-to-door services rendered by CHOs could reduce the incidence of diseases as illustrated:

*“The Scheme must also pay for activities of the CHOs who are supposed to provide health education*. *The health education can lead to change in behaviour and reduce the incidence of disease” We have a formula CHPS plus NHIS equals UHC”* (Stakeholder 18).

*“CHPS which is our primary health care strategy covers about 45% of our health delivery in Ghana*. *They provide health education to people yet their activities are not paid by the Scheme”* (Stakeholder 6).

The fact that NHIS has not made provision to pay for preventive and promotive health services rendered under the Ghana CHPS Strategy was undermining Ghana Primary Health Care (PHC) system. This is because sending clinical nurses to CHPS zones will shift the focus of this system of delivering door-to-door preventive health care to the traditional health facility-based curative health care. This is supported by the illustrative quote from a participant:

*“…*.*because the activities of the CHO does not bring money*, *many district directors prefer to post enroll nurses to CHPS zones because they are clinical assistants and they can prescribe whilst the CHOs sits down in the morning*. *In effect preventive and health promotional activities have declined*. *This will collapse our primary health care system”* (Stakeholder 4).

In addition to that, some participants were of the view that to ensure continuum of care, paying for emergency referral transport system should be considered under the NHIS. The following illustrate this point:

*We need an integrated system for health care*. *In rural areas emergency transport system is a problem*. *So*, *the Scheme should pay for that*. *When the person is referred and does not have money for the transport system*, *he/she will not go to the health facility to be treated for NHIS to pay for the cost of treatment”* (Stakeholder 15).

Some stakeholders were also of the view that NHIS must include cancer treatment as part of the package. The incidence of cancers is increasing and the Scheme is meant to cover common conditions or conditions with higher burden then cancers must be added. It was emphasized that this should include both adult and childhood cancers.

*“In Ghana*, *cancers are becoming a problem and their treatment are not covered by Scheme*. *So*, *they must add cancer because the policy says it must cover common condition in Ghana*. *So*, *if cancers have become common their treatment must be covered by NHIS*. *Go to Korle-Bu*, *you will find evidence of both adult and childhood cancers*. *It is big problem that need to be discussed and added”* (Stakeholder 13).

Stakeholders also highlighted the need for the Scheme to introduce special premiums for people who may wish to insure themselves against the disease conditions outside the benefit package. In their opinion, this could help raise more revenue for the Scheme as illustrated:

*“If I have money and want to pay premium for disease conditions that are currently not covered by NHIS*, *I should be allowed to pay*. *The rich can insure against certain conditions and they must be made to pay more for it*. *This may help raise revenue for the Scheme”* (Stakeholder 5).

Another strategy suggested by stakeholders is the reimbursement of services provided to lower levels of care by consultants through telemedicine. Participants suggested that the Scheme should collaborate with service providers to develop a list of services that can be rendered through telemedicine for consideration. The following illustrate this point:

*“The NHIS should include telemedicine in list of services*. *This will help increase access to consultancy service at lower level and decongest the secondary and tertiary health care”* (Stakeholder 7).

### Financial sustainability of NHIS for Universal Health Coverage

The key sub-themes that emerged from the interviews included; cost containment, increasing funding sources, claims re-imbursement and expanding enrollment.

### Cost containment for NHIS

Despite acknowledging the importance of NHIS in reducing financial burden among the poor and vulnerable population groups, the majority of the stakeholders interviewed were convinced that too many people were exempted from paying the NHIS registration fees and premiums. In their opinion this was one of the threats to the financial sustainability of the NHIS.

*“…*. *Th exception scheme is too broad*. *You have people between 0 to 18 years who are exempted then you have 70 years and above exempted and so you have a small window of people paying premium”* (Stakeholder 6).

Providers’ fraud was also identified as one of the challenges to financial sustainability of the Scheme. Some stakeholders mentioned that providers sometimes submit claims with reimbursement requests for services covered by the NHIS using tariffs that are higher than the official NHIS tariffs. In addition, the claims submitted include services that have not been actually provided. The following illustrate these points:

*“Provider fraud is one of the reasons that makes it difficult for financial sustainability of the Scheme*. *Claims submitted are often more than cost of services provided*. *So*, *the Scheme has to vet the claims to prevent this fraud*” (Stakeholder, 19).

Study participants were also of the view that providers increase cost of providing service to mark-up for delays in re-imbursement of claims submitted to them to cater for inflation.

*“Suppliers increase the cost of items supplied to health facilities to take care of inflations because of delays in payments by the Scheme”* (Stakeholder, 19).

Some stakeholders suggested the government could address this problem through prompt releases of statutory deductions meant for the Scheme as follows:

*“Deduction made as part of Social Security and National Insurance Trust and the NHIS-related taxes on special good and services collected by the Ghana Revenue Authority for NHIS must be sent directly to them*. *However*, *to ensure accountability and transparency*, *an independent fund manager should be established* (Stakeholder 2).

### Expanding funding sources for NHIS

To improve the financial capacity of the Scheme as a sustainability measure, participants suggested the need for new funding sources. Some participants suggested an increase in the National health insurance levy on Value Added Tax (VAT). Others suggested the use of the Heritage Fund from the oil revenue. To some participants, more taxes should be imposed on alcohol and tobacco as their use was responsible for the increasing burden of non-communicable diseases. The following quotes support these assertions from stakeholders:

*“The Scheme has problem meeting its financial obligation to service providers*, *so there will be need for new funding sources such as the Heritage Fund*, *increasing VAT and taxes on tobacco and alcohol*. *The alcohol and tobacco industry are booming in Ghana and these are responsible for increasing levels for diabetes and hypertension in Ghana”* (Stakeholder 12).

Contrary to the view that funding sources for NHIS was inadequate, some participants were of the opinion that the NHIF, NHIL and premium will be enough if financial resources accrued from these funding sources were used judiciously for core NHIS-related activities. The diversion of monies for infrastructure and non-core NHIS activities to them were responsible for the deficits as illustrated below:

*“The 2*.*5% of the VAT and 2*.*5% special levy will be enough for NHIS if the Scheme avoided the waste*. *Go round the region and see the type of buildings they have erected*. *Is that the best way to use monies that is meant to fund health care and help the poor*? *That is why I hold the view that a different agent should be responsible for managing the funds*. *So NHIS can receive the claims*, *vet them and submit to a different agency to do the payment*. *This is the practice in other countries”* (Stakeholder 15).

### Expanding enrollment and active membership for UHC

An obstacle that threatens the operations and sustainability of the NHIS is low active membership. Many enrolled members do not renew their membership when it expires. This reduces the amount that accrue to the Scheme through membership renewal. The following quote illustrate this point:

*“One of the main challenges of the Scheme which serves as barrier to Universal health Coverage is renewal of membership when it expires*. *You see that there is gap between enrollments which is over 60% but active member is less than 40%*. *So*, *we need to address this to increase revenue from renewal”* (Stakeholder 20)

Several suggestions were made in this direction including the alignment of the NHIS to the national health agenda. Participants were also of the view that increasing the number of registered clients in the informal sector could increase NHIS revenue. Enforcing the mandatory registration for all Ghanaians on the NHIS and regular renewal was suggested as an innovative approach to improve financial sustainability and Universal Health Coverage.

*“The Scheme must find innovative ways to capture those in the informal sector*. *if we are talking about Universal Health Coverage*, *it implies that every Ghanaian must be registered and hold an active card at all times”* (Stakeholder 20).

In order to achieve UHC, some participants as well mentioned the need for every Ghanaian to register and remain an active member. This can be achieved by linking mandatory membership to access to certain social services with deterrent measures being denial of such social services. Employers must ensure that all their employees are registered and prospective applicants for employment are active members of NHIS. The following buttress these points:

*“It must be made mandatory and enforced for all employers to ensure that employees are registered with NHIS*. *I am sure if this is already in the law but if not*, *it must be added or enforced if it is already part of the law”* (Stakeholder 15).

*“One way to ensure Universal Health Coverage is to enforce the mandatory requirement for all Ghanaian to enroll in the NHIS*. *So just the like TIN (Tax Identification Number)*, *it must be compulsory for people accessing banking services*, *driver’s license*, *car registration and national identification card to possess valid NHIS card before they are provided with that service*. *People registering their business must be made to make an undertaking to ensure that all employees are registered and have a valid card”* (Stakeholder 3).

### Improving the efficiency of providers’ claims reimbursement

Participants were unanimous in acknowledging delays in reimbursement of claims. These delays were attributed to three main factors; delays in submitting the claims from providers, errors detected during vetting and lack of funds to pay for the service by the NHIS as illustrated by the following quote:

“*The fact that the NHIS reimbursement are not done promptly*, *it has left the health facilities with huge debts and because of that they are finding it difficult to operate*. *One bad thing about NHIS operations that directly affects service delivery is the delays in payment of claims”* (Stakeholder, 8).

The delays were also believed to be due to fewer claims vetting centres. Hence, there is the need to open more claims vetting centres. However, it was noted that some of the delays could be reduced if providers avoided mistakes in completing the claim forms:

*“There is the need for more claim processing centres to be established to speed up the vetting process*. *The vetting sometimes takes a lot of time but sometimes it is due to errors in claims submitted to the Scheme”* (Stakeholder 15).

To reduce the delay, the Scheme introduced an e-claim system where some institutions submit their claims electronically and vetted in the same way. However, this is limited in scope and a scale-up may be necessary.

*“The Scheme has introduced e-claims submission but I think this is limited to a few health facilities*. *If it is scaled-up to cover all health facilities*, *it will reduce the delays to a large extent”* (Stakeholder 3).

### Provider payment mechanisms

All stakeholders interviewed were of the view that the best reimbursement strategy is capitation. This strategy was believed to lead to better quality of health care and capable of reducing provider fraud. The following illustrate these points from stakeholders:

*“Capitation actually improves service delivery and quality of care because people who are put in that place demand for the services to be better and reduce fraud in the submission of claims”* (Stakeholder 8).

However, participants emphasized the need to enforce the gatekeeper system to make capitation successful as illustrated:

*“…*. *So*, *we should go back to capitation and limit it to primary health care facilities and enforce the gatekeeper system*” (Stakeholder 7).

### Governance and transparency in the operations of NHIA

Interviewees were of the view that NHIS should remain an agency under the Ministry of Health. The creation of the deputy Chief Executive Officer and other divisional heads was meant to improve the governance structures of the Scheme. However, participants were of the view that the Scheme has overly been politicized. This over-politicization was therefore hampering the smooth running of the Scheme. This was unanimous among interviewees as they indicated the urgent need for the Scheme to be protected against political interference. The following quotes illustrate these points:

*“The NHIA should remain as an agency under the Ministry of Health but it must work closely with Ghana Health Service and other service providers*. *The main issue about the Scheme is the political interference which is clearly undermining the operations of the Scheme*. *So*, *the Scheme must be insulated against political interference”* (Stakeholder 10).

*“We have over-politicized the Scheme making it difficult for it to operate as independent agency*. *Monies meant for NHIS are sometimes diverted for other activities whilst claims remain unpaid for months*. *Going forward we need to de-politicize the Scheme”* (Stakeholder 2).

Participants were of the view that the frequent change in Chief Executive Officer of NHIA interferes with the governance structure of the Scheme. The Chief Executive Officer (CEO) is often appointed by the sitting president of Ghana and changed anytime there is a change in government. These appointments, to the study participants, breed political interference making it difficult for the Scheme to be independent. According to the participants, this in the past has resulted in diversion of NHIS funds for other activities reducing the financial capacity of Scheme and delays in re-imbursement. This is supported by the illustrative quote:

*“The president appoints the Chief Executive Officer and other senior members of the Scheme*. *So*, *they are forced to follow whatever the appointing authority wants*. *So*, *you see sometimes*, *monies meant for the Scheme are used for other government activities whilst service providers’ claims remain in arrears for months”* (Stakeholder 17).

Another governance challenge identified in the study is weak human resource capacity of NHIS district offices and vetting centres. Some participants were of the view that NHIS is not well resourced at the district level who are at the forefront of the operations of the Scheme. This according to some participants results in delays in claims processing.

*“The Scheme has human resource challenges at district level*. *This results in delays in the claim processing*. *Some of the people employed are not technical people*. *The political interference sometimes results in appointment of party members who may not have the technical know-how to manage the Scheme”* (Stakeholder 19).

Participants were also of the view that the breadth of NHIS-related activities undertaken by the Scheme was too broad, creating transparency challenges. The Scheme is in-charge of credentialing health facilities, vetting of claims, reimbursement, and registration of beneficiaries. Participants were of the view that some of these activities should be relayed to other institutions. To improve transparency, participants were also of the view that the credentialing of facilities should be left to the Health Facility Regulatory Authority (HeFRA).

*“…*. *The other thing is credentialing*, *that function should be done by Health Facility Regulatory Authority (HeFRA) and not the current where the NHIS”* (Stakeholder 10).

Furthermore, other agencies should be created and given some of the responsibilities of vetting of claims and reimbursement. NHIA and NHIS must be in-charge of the registration and provide oversight in the implementation of Scheme. However, the Scheme currently is in-charge of vetting the claims at the same time reimbursement. This in the opinion of some stakeholders allowed for the abuse of the system. The following illustrate these points:

*“NHIA seem to be a referee and a player*, *there is no regulator because nobody regulates NHIA and so they have the whole system to themselves*. *You know in other countries*, *those who compile the claims are not the ones to do the payment*, *there are different bodies responsible for various activities but for Ghana*, *the NHIA does everything*. *They do the vetting*, *they do the payment and everything and there is no independent verification that goes on*, *the same people (NHIA) go and verify*, *they take a decision*, *they deduct and they are not regulated and I think that is very wrong”* (Stakeholder 3).

It was suggested that an independent body should be in-charge of determining the tariffs for services rendered to card holders. This in their view will resolve the current trust issues between the Scheme and providers. Providers were of the view that the tariffs are low whilst the Scheme held a contrary view. To foster transparency, participants suggested an independent institution should be created in-charge of determining and reviewing tariffs. The following quotes illuminate these points by participants:

*“There should be an independent institution in-charge of determining and reviewing tariffs*. *The Scheme claims the tariffs are realistic whilst service providers think otherwise*. *This has often resulted in conflict*. *So*, *an independent institution conducting the review will improve transparency”* (Stakeholder 18).

*“The tariffs should also be reviewed frequently to cater for inflations*. *The law requires a frequent review-very year but this is not always done*. *We providers struggle to break even with the low tariffs for the service we render”* (Stakeholder 7).

## Discussion

The qualitative study was conducted to identify how to position the NHIS for financial sustainability to ensure Universal Health Coverage. Key stakeholders in this study acknowledged that the Scheme in recent time faced challenges in meeting its financial obligations. To sustain the Scheme, stakeholders highlighted the need for quick releases of statutory deductions for Scheme and advocating for new funding sources. Frequent review of tariffs was identified as critical to cater for inflation and introduction of capitation as provider payment mechanism to reduce fraud. In addition, inclusion of preventive and promotive health services was deemed relevant to align the Scheme with Primary Health Care system. In view of the changing epidemiology in Ghana, it was suggested that new health conditions be included into the list of insured conditions to position the Scheme for Universal Health Coverage.

Geographical and financial access are important and this has been catered for by CHPS and NHIS respectively. Hence to ensure that these two approaches are properly aligned, it would be important for NHIS to pay for the preventive service, as envisioned in the Primary Health Care declaration. Otherwise, the CHPS strategy stand the risk of collapsing as focus may shift to curative health care instead of preventive and promotive health care. Currently, parallels exist between PHC principles embedded in CHPS and NHIS provisions on reimbursement of claims. Preventive and promotive health care which form the tenet of PHC and CHPS are not reimbursable in NHIS. Given the success of CHPS in reducing maternal and child health morbidities [38,41,42] and uptake of reproductive health services [43], it would be important to design a package for the work of CHOs (Community Health Officers) who currently work in the CHPS zones. Their activities can reduce the burden of diseases in communities and has the potential of reducing the OPD attendance which will invariably reduce the number of claims submitted to NHIA. Beyond the NHIS for health financing, families and communities must be strengthened to address preventive as well as associated health care costs to be able to reduce disease burden among Ghanaians to achieve SDG 3 [37].

Another aspect of care along the continuum is the referral system. However, NHIS is yet to cover the cost of emergency transport, and most communities have no system for emergency obstetric and neonatal care. This will also require critical review to remove delays in emergency referrals. CHOs may not have the expertise to provide emergency obstetric care but may provide first aid and refer to the next level of care. Transportation cost may still serve as a barrier in some instance therefore denying the individual access to the free midwifery and delivery services to reduce maternal mortality. Paying for the referral system will support the synergies and linkages between the three-tier referral system; community level, the sub-district level and district hospital/health administration levels to provide appropriate quality and continuum of health care.

Again, the increasing burden of cancers led to stakeholders recommending that treatment for cancers should be added to the package for NHIS. Majority of cancer medication are not included in the NHIS medicine list. However, the records show an increasing incidence of cancers [44] in Ghana which will require anticancer medications. Currently cervical and breast cancer management are covered by NHIS. However, management for cancers among children is not covered by the NHIS. This therefore deprives children who have been identified as exempt group for NHIS registration from cancer management. Medicines for managing cancers should be given critical consideration for inclusion in NHIS registration because of emerging threats of cancers in Ghana.

At present NHIS allows for visiting consultants to facilities at lower level to provide health care which is reimbursable. With increasing technology, another area of consideration by the Scheme should be telemedicine which has the potential of increasing access to tertiary health care at lower facilities. Telemedicine also has the potential of reducing congestions at the higher-level facilities as well as the inconvenience in accessing tertiary health care. This is especially important for chronic disease which often require specialists or consultants who may not be available at the lower level. Telemedicine have been found to increase access to specialist care in rural areas [45,46] and improving Universal Health Coverage [47]. Early specialist management of chronic disease have been found to improve prognosis and reduce cost of managing complications [48,49]. Going forward it would be important for service providers and NHIA to develop separate costing Scheme for telemedicine.

The study also underscores the need for incorporation of annual medical examination for subscribers. The package for the examination can be discussed by stakeholders and accepted. However, to be able to implement such policy, it requires standardization of the medical examination and disaggregated by age and sex to capture peculiar risk factors. Costing of the minimum package for medical examination can be done for reimbursement. The findings suggest that it could cover emerging non-communicable diseases such as hypertension, diabetes and cancers and their risk factors. Since many people may be unwilling to visit health facilities regularly for medical examination because of issues related to cost, making it part of the benefits of the NHIS will compel people to undergo annual medication examination. Some of these diseases when detected early are manageable but at advance level may require more advanced medical expertise to manage which may cost the Scheme more money.

The findings show that delays in claims reimbursement and providers’ fraud was undermining the financial sustainability of the Scheme. To circumvent the delays and provider fraud, capitation payment system was adopted. Capitation was piloted in Ghana in the Ashanti region. However, its implementation was bedeviled with several operational challenges, hence it could not be scaled-up. Nevertheless it has reported that capitation increased the trust among providers and clients [24] as well reduce providers’ fraud [50–52]. Some participants are of the view that capitation could better be piloted in rural settings and limited to primary health care (PHC) structures. Lessons learnt could then inform nationwide scale-up. There is therefore the need to re-pilot capitation in rural area and limiting it to PHC facilities. Capitation has been widely reported as the strategy that propelled Thailand towards Universal Health Coverage [53] and viewed as better payment policy in a study conducted in South Africa [54]. However, gatekeeper system is indispensable in its effective implementation [55,56].

The institutional arrangement of the NHIS makes it an agency under the Ministry of Health with the President appointing the Chief Executive. This arrangement according to the study findings affects the smooth running of the Scheme as it is subject to political interference. To ensure good governance, it will be important to insulate the Scheme from political interference. The country’s constitution and democratic mechanisms dictate the transparency of decision-making processes and thereby determine the degree to which politicians are held accountable [57]. The control of the Scheme by government has in the past resulted in diversion of NHIS funds into other government activities other than core mandate of the Scheme [58]. This practice negatively affects the finances of the Scheme with implications for sustainability.

## Conclusions

The Scheme could potentially achieve UHC if protected from political interference to improve the governance and transparency that affects the finances of the scheme and the expansion of services to include preventive and promotive services and cancers.

## Supporting information

S1 File (DOCX)

## Abbreviations

CHOs: Community Health Officers
CHPS: Community-based Health Planning and Services
NCDs: Non-Communicable Diseases
NHIA: National Health Insurance Authority
NHIS: National Health Insurance Scheme
OOP: Out-Of-Pocket Payment
PHC: Primary Health Care
UHC: Universal Health Coverage
