# Supplementary material for: Positioning the National Health Insurance for financial sustainability and Universal Health Coverage in Ghana: A qualitative study among key stakeholders
Source: PLoS One. 2021 Jun 15;16(6):e0253109. doi: 10.1371/journal.pone.0253109 (PMC8205146; doi:10.1371/journal.pone.0253109)
Supplement: S1 File — (DOCX) [file pone.0253109.s001.docx]

Stakeholders identified and interviewed

| Stakeholder | Legitimacy | Resources | Network | Influence and power | Key stakeholder or Actor  Yes/No |
| --- | --- | --- | --- | --- | --- |
| National Health Insurance Authority | √ | √ | √ | √ | Yes |
| Social Security and National Insurance Trust | √ | √ | √ | √ | Yes |
| Ministry of Finance | √ | √ | √ | √ | Yes |
| Ministry of Health | √ | √ | √ | √ | Yes |
| Ghana Health Service | √ | √ | √ | √ | Yes |
| Christian Health Association of Ghana | √ | √ | √ | √ | Yes |
| Ministry of Gender and Social Protection | √ | √ | √ | √ | Yes |
| Ghana Medical Association | √ | √ | √ | √ | Yes |
| Ghana Registered Nurses and Midwives Association | √ | √ | √ | √ | Yes |
| Coalition of NGOs in Health Chairperson | √ | √ | √ | √ | Yes |
| Pharmaceutical Society of Ghana | √ | √ | √ | √ | Yes |
| Private Health Providers Association | √ | √ | √ | √ | Yes |
| Trade Union Congress | √ | √ | √ | √ | Yes |
| Employees of Private Sector | √ | √ | √ | √ | Yes |
| Association of Health Service Administrator of Ghana (AHSAG) | √ | √ | √ | √ | Yes |
| Civil Society Organization | √ | √ | √ | √ | Yes |
| Parliamentary Select Committee on Health | √ | √ | √ | √ | Yes |
| Social Welfare Department | √ | √ | √ | √ | Yes |
| UNICEF | √ | √ | √ | √ | Yes |
| WHO | √ | √ | √ | √ | Yes |

**STAKEHOLDERS ANALYSIS AND MAPPING QUALITATIVE INTERVIEWS**

**STAKEHOLDER CHARACTERISTIC EXTRACTION SHEET**

| No | Name of Institution | Rank of Stakeholder in Institution | Years of Service | Sex | Interest |
| --- | --- | --- | --- | --- | --- |
|  |  |  |  |  |  |
|  |  |  |  |  |  |
|  |  |  |  |  |  |
|  |  |  |  |  |  |
|  |  |  |  |  |  |
|  |  |  |  |  |  |
|  |  |  |  |  |  |
|  |  |  |  |  |  |
|  |  |  |  |  |  |
|  |  |  |  |  |  |
|  |  |  |  |  |  |
|  |  |  |  |  |  |
|  |  |  |  |  |  |
|  |  |  |  |  |  |
|  |  |  |  |  |  |
|  |  |  |  |  |  |
|  |  |  |  |  |  |
|  |  |  |  |  |  |
|  |  |  |  |  |  |
|  |  |  |  |  |  |

**R4D PROJECT: DOCUMENTATION OF THE POLICY CHANGES IN THE NATIONAL HEALTH INSURANCE SCHEME AND LIKELY EFFECTS ON SUSTAINABILITY OF THE SCHEME**

**INTERVIEW GUIDE FOR STAKEHOLDERS FOR NHIS POLICY REVIEW**

Opening

1. What is your organization’s interest in social and inclusion and universal health coverage

There are currently ongoing debates on NHIS and likely policy changes

**Views about NHIS**

1. What are your opinion about the NHIS in Ghana?
2. In your view, what interest has the NHIS served?
3. Over the 10 years of implementation of the NHIS, what changes will you suggest to be made in NHIS in Ghana?
4. How is the NHIS positioning itself to meet universal health coverage?

**Beneficiaries and Package**

1. What services should be included in the benefit package in the new policy?
2. Who should be excluded in the new benefit package?
3. How protective should the new benefit package be to the poor?
4. How does it control for moral hazard?
5. What provider mechanism should be adopted for reimbursement of claims?
   1. Who bears the risk of choice
6. What would be the role and interest of health care groups in the new benefits package?

**Sustainability**

1. What measures have been put in place to ensure sustainability of NHIS?
2. What are the ongoing changes being made in the NHIS that may affect social inclusion?
   1. How can these ongoing changes affect sustainability of the scheme?
3. What is your views about the NHIS funds being paid to the consolidated funds?
4. What new funding sources of NHIS would you suggest to improve financial capacity of the scheme?
5. What can be done to minimize delays in reimbursement of claims?
6. What are your views about the pricing of medicines by NHIS?

**Governance**

1. What is your view about the current governance structure of the NHIS?
   1. What is your view about the NHIA reporting to Ministry of Health?
   2. If you had the opportunity of reviewing NHIS policy, what governance structure will you suggest for the NHIA?
2. What is your views about the argument that NHIA should be restructured to report to the Minister of Finance and Economic Planning

**Closing:** Are there any other things you will like to share with us regarding, operation of NHIS to help improve governance, transparency and sustainability?
